# What's Up With These Conversational Health Agents? From Users' Critiques to Implications for Design

**DOI:** 10.3389/fdgth.2022.840232

**Published:** 2022-04-07

**Authors:** Raju Maharjan, Kevin Doherty, Darius Adam Rohani, Per Bækgaard, Jakob E. Bardram

**Affiliations:** ^1^Department of Health Technology, Technical University of Denmark, Lyngby, Denmark; ^2^Department of Applied Mathematics and Computer Science, Technical University of Denmark, Lyngby, Denmark

**Keywords:** conversational user interface (CUI), conversational agent (CA), voice user interface (VUI), virtual assistant (VA), Alexa, health and wellbeing, text analysis, structural topic model (STM)

## Abstract

Recent advancements in speech recognition technology in combination with increased access to smart speaker devices are expanding conversational interactions to ever-new areas of our lives – including our health and wellbeing. Prior human-computer interaction research suggests that Conversational Agents (CAs) have the potential to support a variety of health-related outcomes, due in part to their intuitive and engaging nature. Realizing this potential requires however developing a rich understanding of users' needs and experiences in relation to these still-emerging technologies. To inform the design of CAs for health and wellbeing, we analyze 2741 critical reviews of 485 Alexa health and fitness Skills using an automated topic modeling approach; identifying 15 subjects of criticism across four key areas of design (functionality, reliability, usability, pleasurability). Based on these findings, we discuss implications for the design of engaging CAs to support health and wellbeing.

## Introduction

Recent advancements in speech recognition technology in combination with the growing popularity of smart speaker devices, including Amazon Echo and Google Nest have made it increasingly possible to engage with Conversational Agent (CA)s in the many and varied contexts of our everyday lives, for purposes including to support our health and wellbeing. According to one recent survey (n = 1004) 52.0% of U.S. adults possess an interest in the use of CAs for healthcare, while 7.5% have already used such systems for a healthcare-related task, such as inquiring about symptoms of illness, searching for information concerning medication usage, or seeking care and treatment options (1).

This has led in turn to increased commercial and research interest in these systems' potential to support wellbeing (2–6). A recent review by Chung et al., for example, reveals an upward trend (from less than 175 Skills in December 2016 to more than 275 by April 2017) in the total number of CAs published for health and wellbeing purposes *via* Amazon Alexa alone since the release of their Software Development Kit (SDK) in June 2015 (7). And, research suggests that CAs do have the potential to support users' wellbeing by serving as convenient and easy to use tools facilitating self-care, expanding access to health-related information, supporting health tracking and monitoring, and providing data to aid decision-making (8)—for users with physical (9), sensory (10), and cognitive impairments (11) in particular.

Despite the growing popularity and adoption of these emerging systems, however, there exist also many outstanding challenges impacting the present and future use of these systems for health- and healthcare-related tasks. Challenges previously identified by researchers include; misinterpretation and misunderstanding of user utterances resulting in confusion and further errors (12–14), the need for users to know in advance what they can or cannot say during interaction (9, 15, 16), and CAs' often monotonous, robotic and unnatural vocal features negatively impacting users' engagement (17–20). Evidence also exists, however, to suggest that appropriate interaction design choices can effectively shape interaction with these less-than-perfect technologies in order to improve both their usability and user experience (21–25).

In line with such efforts to develop continuous improvements in the design of CAs and to realize the full potential of these nascent systems to support health and wellbeing, researchers have begun to explore the impact of diverse conversational designs on self-report behaviors and experiences (26), self-reflection and learning during the self-report of mental and physical health and wellbeing (27, 28), users' preferences in relation to emotional interaction (29), and the impact of random back-channeling on user engagement (22). Such study results are key to supporting improvements in the design and deployment of CAs for health and wellbeing (Conversational Health Agents (CHAs) hereafter). Most research to date has however, focused on developing user requirements or understanding novel interactions in lab-environments, taken place in specialized healthcare contexts, or been conducted with narrowly-defined groups of users, technology designs and system configurations. We therefore know less about users' experiences in relation to the real-world and voluntary adoption of the diverse CHA designs currently available through the commercial stores.

Such an holistic understanding of users' needs and experiences is essential to identifying new design opportunities, helping designers to effectively allocate time and resources toward the features which matter most, and understanding the current breadth and depth of design choices in support of more effective and engaging systems. With the aim of informing CHA design to support wellbeing, this article addresses the following research questions;

RQ1: What are the primary subjects of critique raised by users in relation to the state-of-the-art CHAs?
RQ2: Given users' concerns, which choices might designers make to improve the current design(s) of CHAs?

To answer these research questions, we analyze a large data set comprising 2741 critical reviews of 485 Skills published within Amazon Alexa's health and fitness category. We employ an automated topic modeling technique to identify the key subjects of critique across these reviews and describe users' pain points according to Aarron Walter's hierarchy of users' needs as pertaining to considerations of functionality, reliability, usability and pleasurability (30).

Based on our results, we then present implications for the design of CHA experiences rendered; (i) functional by keeping setup processes simple, facilitating connectivity, and preparing meaningful responses to predictable queries; (ii) reliable by providing ancillary support, and fostering user-vendor relationships; (iii) usable by supporting navigation and personalization features; and (iv) pleasurable by prioritizing brevity, providing variety, approaching voice as design material, and commercializing ethically.

This work contributes; (i) an understanding of critical factors affecting users' experiences of CHAs, (ii) implications for the future design of effective and engaging CHAs, and (iii) a novel approach to the critical analysis of online reviews in support of design implications.

## Related Work

Human-Computer Interaction (HCI) researchers have traditionally employed a wide variety of methods from observational lab and field studies to user-centered interviews, focus-groups and questionnaires as means of generating knowledge of users' needs, values and experiences of designed systems throughout the product lifecycle. These methods offer rich insight into a specific user group's experiences of particular application and service designs, although are also human-resource intensive, requiring considerable time to plan and conduct. A recent review of the HCI literature concerning speech interfaces suggests a lack of empirical work in voice-based conversational interaction due to the barriers to evaluation in real-world contexts, thereby impairing our understanding of CA user experience (31). As an alternative and complementary means of obtaining a broad overview of diverse and representative users' experiences of a wide variety of system configurations in the real-world, researchers have therefore also turned, more recently, to analysis of online content including customer reviews provided through commercial sites such as amazon.com—as self-reports of end-users' experience made in the wild, and in their own words (32).

As shown in Table 1, online reviews have been used in previous research to learn about users' perspectives on Alexa's health and fitness skills as well as various aspects of the Alexa devices.

**Table 1:** Summary of the results from prior related work analyzing reviews of Alexa health and fitness skills and Alexa devices.

**Authors**	**Review of**	**# of Reviews**	**Method**
Pradhan et al. (9)	Amazon Echo, Dot & Tap devices	346	Manual
*Major Outcomes:*			
1. Novel use cases of the Echo device for people with disabilities, including in speech therapy and as supports for caregivers.			
2. Challenges for people with disabilities include, discoverability (particularly for users with visual impairments) speech recognition, device ecosystem related issues (need to ensure that the entire device ecosystem–and not just the voice interaction–is accessible), memory demands (having to remember voice commands is problematic for older adults or users with cognitive impairments).			
Purington et al. (33)	Amazon Echo & Tap devices	587	Manual
*Major Outcomes:*			
1. The degree of device personification is linked with the sociability of interactions. Personification predicts user satisfaction.			
2. Reviewers mentioning multiple member households are more likely to personify the device than reviewers mentioning living alone.			
Shin and Huh-Yoo (34)	Health and Fitness Skills	443	Manual
*Major Outcomes:*			
1. Skills are primarily used as a way to jump start behavior change, to enhance health and wellness routines, and to overcome time and spatial limitations.			
2. Trust in the content provider and transparency of the CHAs' limitations are important factors for users' adoption of these systems.			
3. Design considerations: matching initial user expectations, transparency concerning CHA's limitations, methods of commercialization, use cases for peripheral devices and the quality of agents' instructions and commands.			
4. In addition to Nielsen's design heuristics, authors recommend increased personification of agents, provision of content from trusted sources, and allowing users to tailor utterance speed and tone among other CHA characteristics.			
Gao et al. (35)	Amazon Echo devices	55502	Automated
*Major Outcomes:*			
1. Reviews reflecting personification of Echo displayed more positive emotions than those which considered Echo solely an electronic device.			
2. Echo features liked by users: hands-free use, efficient task management (e.g., shopping lists, timer and alarm settings, ordering from Amazon) and information retrieval, entertainment, and connectivity with other smart home systems.			
3. Echo features disliked by users: voice/language (problems misinterpreting speech or returning inappropriate responses), privacy-related measures (always-on listening), device limitations (query only, English-only support, the inability to change the wake word, lack of portability, the need to be connected, upgrade notifications at midnight, and the inability to reschedule updates), as well as application limitations (support for only 1 timer and 1 alarm, the inability to fast forward or skip ahead when listening to music)			

For example, Pradhan et al. (9) studied 346 online reviews of Amazon Echo, Dot, and Tap devices to understand accessibility issues encountered by users with disabilities, their general experiences and suggestions for the improvement of voice-controlled Interactive Personal Assistants (IPAs). By manually analyzing reviews describing the use of the device by a person with a cognitive, sensory, or physical disability, written from either first- or third-person perspectives, the authors identified several novel use cases for these devices, including in speech therapy and as supports for caregivers, and suggested the need to design Skills to support discoverability in particular as a primary concern for users with visual impairments. Similarly, Purington et al. (33) analyzed 851 reviews of the Amazon Echo device to understand the degree to which user reviews touch upon the importance, for users, of device personification, sociable interaction, and factors influencing user satisfaction. Results suggested that greater personification of the device is correlated with increased social interaction, and that personification predicts user satisfaction.

Shin et al. (34) analyzed 433 user reviews of Amazon Alexa's health and fitness skills in an effort to identify users' perceptions of the strengths of CHAs for everyday health and wellness management, and to develop design heuristics to support the future development of CHAs. Findings from this study revealed the importance of trust in the content provider and transparency in regard to CHAs' limitations to users' adoption of these systems, while also highlighting CHAs' capacity to enable people to overcome logistical barriers to improving their daily health and wellness routines. Based on these findings, the authors extend Nielsen's design heuristics (36); recommending increased personification of agents, the provision of content from trusted sources, and allowing users to tailor utterance speed and tone among other CHA characteristics, in support of the design of these systems to support health and wellbeing.

Results of these studies are often limited by small sample sizes however, and advancements in computer-based topic and text analysis techniques have allowed researchers to begin analyzing also the growing availability of text data, in order to discover hidden topics and themes. For example, Gao et al. (35) employed Natural Language Processing (NLP) techniques to support analysis of 55502 Amazon Echo reviews in order to understand just how users personified the device (e.g., as a spouse, friend, or pet), finding that those reviews reflecting personification of Echo displayed more positive emotions than those which considered Echo solely an electronic device. More recently, advancement in unsupervised machine learning-based topic modeling techniques such as Latent Dirichlet Allocation (LDA) (37), Correlated Topic Model (CTM) (38), and Structural Topic Model (STM) (39) have allowed researchers to analyze mounting availability of text data to discover hidden topics and themes efficiently and effectively. For example, Hu et al. (40) analyzed online customer reviews from hotels.com to understand customers' dissatisfaction across different grades of hotels on various aspects. McInerney et al. (41) explored how palliative care is understood in the context of dementia from online posts. Tvinnereim et al. (42) analyzed open ended survey data to understand public perceptions of air pollution and climate change.

These studies provide initial evidence of the value of both manual and automated analysis of online reviews as means to understand users. The Alexa health and fitness Skill domain likewise caters to a wide variety of user needs and values although with a shared purpose in mind—to support health and wellbeing—leading to diverse applications to support tasks from health-related information queries to self-monitoring exercises and behavior change activities.

This article extends and builds upon prior research in three primary respects; by (i) analyzing a significantly larger and more inclusive sample of reviews (*n* = 2741) reflecting users' experiences of a wider variety of health and fitness Skills (485 Skills from 19 categories), (ii) focusing on critical reviews as a particularly informative source of insight pertinent to design, and (iii) employing an automated topic modeling approach enabling a broad mapping of the design space, and the consistent analysis of users' critiques and experiences as expressed through online review.

## Methods

In order to develop knowledge of users' experiences, and in particular critiques, of current and diverse publicly-available state of the art CA systems for health and wellbeing, we turned to analysis of a large sample of critical reviews of Amazon Alexa's health and fitness Skills. We chose to focus on the Alexa system as Amazon holds the largest market share of smart-speaker devices and provides a Health Insurance Portability and Accountability Act (HIPAA)-compliant solution for Alexa and custom Skills making it the preferred platform for publishing CHAs (1).

Our approach entailed, to the best of the authors' knowledge, the first application of STM (39) to a large body of Amazon Alexa's health and fitness Skill reviews. STM is a generative model of word counts, which assumes that “a topic is defined as a mixture over words where each word has a probability of belonging to a topic. And a document is a mixture over topics, meaning that a single document can be composed of multiple topics” (39). We use STM for this study as it offers features such as topic discovery, text preprocessing, model search and validation, and topic visualization in one place, which enable researchers to analyze text corpora and draw inferences efficiently and effectively.

### Data Collection

On August 14, 2021, Amazon's Alexa Skill catalog was searched identifying 1093 Skills matching the following inclusion criteria; (i) designed for the English language, (ii) listed under the health and fitness Skill category, and (iii) having received at least one customer review^1^. A custom Python (Version 3.9.6) script was then employed to first extract the Amazon Standard Identification Numbers (ASINs) of the Skills and then the following data for each Skill; name, description, average star rating, and critical reviews. We focus on critical reviews as primary means of insight into the critiques, leveraged by users, toward the capabilities and design of these Skills. According to Amazon's criteria, a user review is considered ‘critical' when assigned less than four stars out of five. This process resulted in a sample of 3117 critical reviews provided in regard to 556 Skills in receipt of at least one critical review.

### Skill Categorization & Review Selection

To facilitate our analysis, we next classified each of these Skills according to one or more of the 25 health and wellbeing Skill categories previously developed by Chung et al. (7). The protocol for this categorization process entailed the independent classification of each Skill by two reviewers according to the title and description of the Skill provided by the vendor. When reading of both title and description was insufficient to permit classification of the skill, user reviews were then examined. The description provided for a Skill titled “Deepak Chopra”^2^ for example, read only “Deepak Chopra,” and subsequent examination of users' reviews revealed the Skill to concern “meditation” and “motivation.” Those skills which appeared unrelated to health and wellbeing despite their marketplace classification were categorized as “other” (e.g., Country Girl Hemp^3^, Red Dragon News^4^). Inter-coder agreement for this process, as calculated by Fleiss's Kappa (κ = 0.8), was substantial (43). Disagreements between coders were reconciled through discussion until consensus was reached concerning the classification of all Skills according to one or more categories.

Following this process, and in order to ensure our analysis was focused only on those Skills designed to support health and wellbeing, we excluded all Skills pertaining to the categories “air quality and environment monitoring,” “Baby naming,” “Dog monitoring and tracking,” “Global positioning system or geographic information system,” and “other,” as well as any duplicate reviews from further analysis—resulting in a revised sample of 2963 reviews of 509 skills.

Following the common STM practices (39), we pre-processed the sample using Quanteda (Version 2.1.1) (44) which offers a much richer set of functions than the built-in textProcessor function (39). Pre-processing of the sample included (i) stemming the wording of each review to its root form (e.g., relaxed and relaxation reduce to relax), (ii) normalization by transformation to lower-case, and (iii) removal of punctuation, numbers, symbols, English-language stop words (e.g., words such as “this,” “is,” “a,”) words appearing fewer than 10 times in the corpus, and the words “alexa” and “skill” as there is little information added from these words, and the computational cost of including them in the model can be substantial (39, 45). Finally, we generated a Document Term Matrix (DTM) ordering each document (review) as a column, and each word as a row in the matrix (46). In the process, empty documents were dropped, resulting in a final data set comprising 2741 reviews of 485 skills.

### Structural Topic Model (STM)

Following these initial stages of pre-classification and pre-processing, an STM was then generated on the resulting DTM, using the STM package (version 1.3.5) in R (version 3.6.2) for topic modeling (47).

The STM estimates topics based on a pre-specified number of topics (*K*). According to the authors of STM, there is no “right” answer to the number of topics appropriate for a given corpus (47, 48), and no statistical tests to determine the optimal number of topics for a model nor the quality of those topics (49). STM does however support numerous diagnostic techniques for estimating the number of topics, including residuals (50), semantic coherence (51) and exclusivity analyses (52).

**Figure 1:**
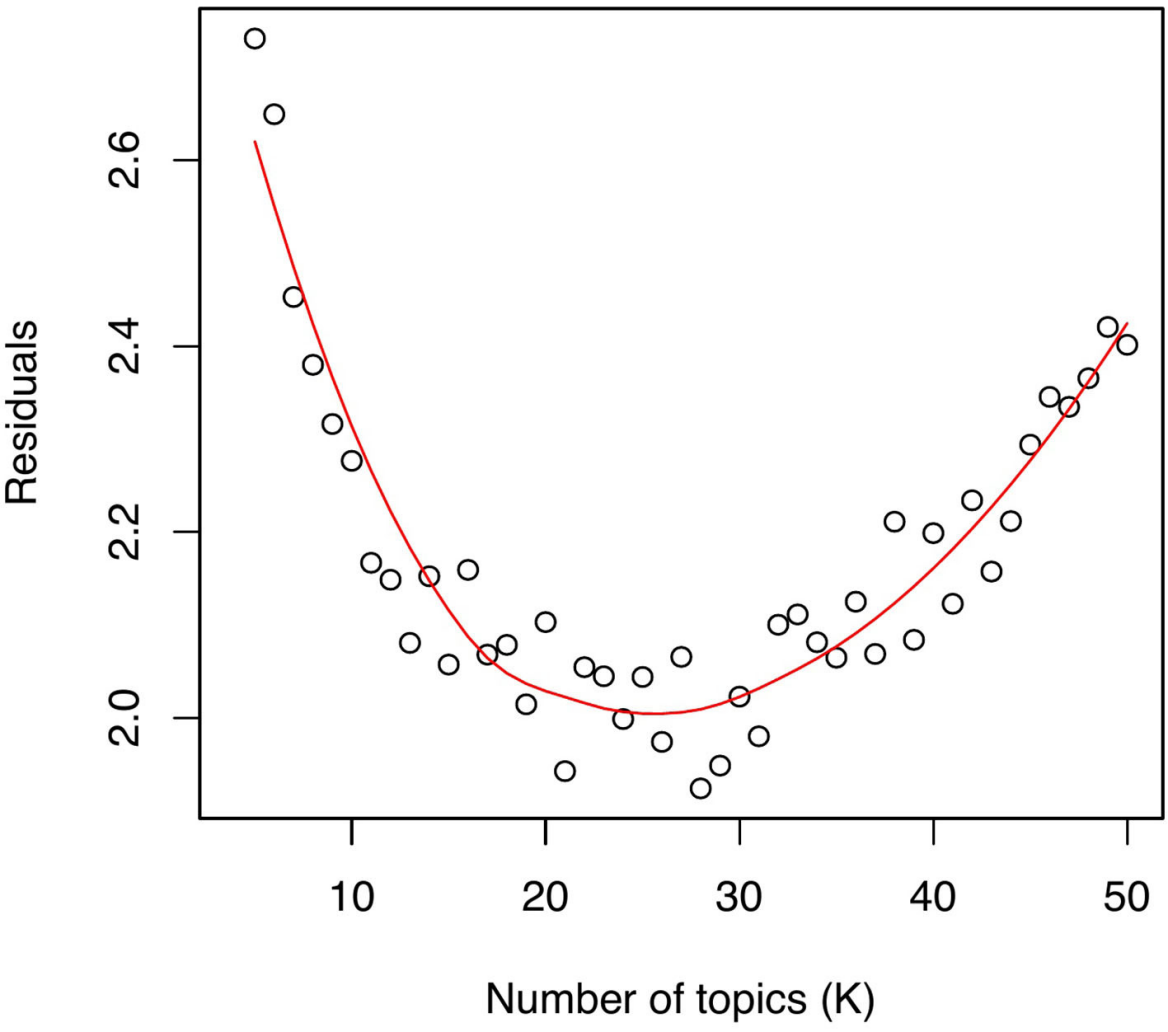
Diagnostic values of the residuals across the models with a value of *K* ranging from 5 to 50.

In this study, we employed a mixed methods approach to identifying the optimal number of topics (*K*), inspired by related work adopting a similar approach to analyzing Twitter data using STM (53). We began by estimating the model fit by comparing the residuals of the models with values of *K* ranging from 5 to 50, as recommended by the authors of the STM for a corpus size ranging from a few hundred to a few thousand documents (47). We then examined the diagnostic values of the residuals, semantic coherence, and exclusivity of each of the estimated models (see Figure 1), selecting ten candidate models with a value of *K* ranging from 20 to 30. These models contained low residuals—representing distances between observed and predicted values—and therefore best approximating the text of the reviews.

We next examined the semantic coherence (51) and exclusivity (52) of the individual topics of each of these candidate models using STM's topicQuality function.^5^ Semantic coherence is a measure of the probability that a set of topic words^6^ co-occur within the corpus, and exclusivity refers to the probability that the top words representing the topic do not appear as top words for other topics.

While these measures are efficient, researchers' judgment of topics by close reading of example documents is essential in the selection of the optimal STM model (39). Therefore, two authors performed an additional manual examination of the collections of top words (highest probability and FRequency & EXclusivity (FREX) words) and reviewed five reviews highly associated with each candidate model's topics^7^ (39). The highest probability words represent a topic's semantic coherence and tend to co-occur in other topics, whereas FREX words are weighted by their overall recurrence and exclusivity to a topic. On the basis of these two criteria, we selected the model with 22 topics for further analysis.

#### Topic Interpretation & Model Validation

Examining the selected model, the first author then analyzed the top words and 20 associated reviews for all topics, labeling each with a phrase communicating their common meaning. A topic comprising the top words “link,” “account,” “disappoint,” “amazon,” “headspac,” “unabl,” and “useless” for example, in addition to the exemplar review “*Unable to link to my account,”* was given the topic label “account linking.” Each topic label was iteratively reviewed and refined with the additional involvement of the second author in order to produce the final set of topic labels.

Following this initial labeling stage, topics critiquing the same CHA features were grouped. Topics referencing subscriptions, including frequent subscription prompts, subscription upselling, and subscription cancellation, were grouped into a single topic entitled ‘commercialization methods' for example. During this phase of the process we additionally compared our results with Shin et al.'s prior work (34), finding that the topics identified encompassed and expanded upon each of the themes identified through their research, in turn confirming the external validity of our results.

Aarron Walter's hierarchy of user needs was finally employed to structure and facilitate categorization of each of these topic groups based on their intuitive conceptual similarities (30). We chose this model to enable us to understand areas of users' pain points in relation to their use of the CHAs and to map these according to users' needs in order to help designers allocate their time and resources toward the features which matter most to reviewers. Topic groups pertaining to issues with CHA functioning (e.g., problems enabling skills or logging in, account linking, and CA–user misunderstandings), for example, were categorized under the “functional” category.

## Results

Seeking broad understanding of users' critical experiences of Amazon Alexa health and wellbeing Skills, we turn then to analysis of a structural topic model comprising 22 topics representing 2741 reviews of 485 Skills across 19 health and fitness categories.

### Mapping the Skills Space

**Figure 2:**
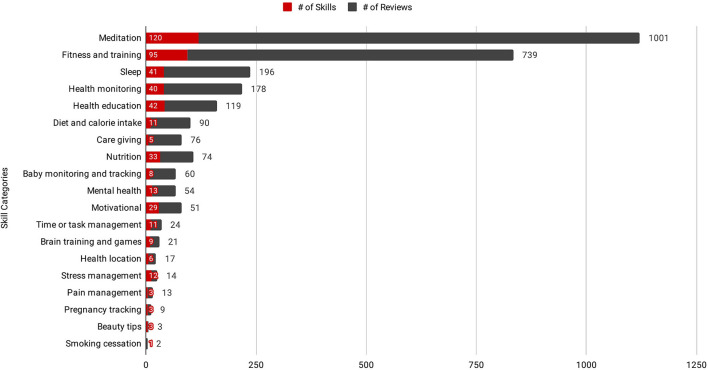
Distribution of Skills and reviews for each of the 19 health and fitness categories included in this study.

Examining first the distribution of Skills across these 19 health and fitness categories, we see clearly an increased prevalence of both reviews and Skills relating to meditation and fitness training (see Figure 2).

### Making Sense of Skill Reviews

**Figure 3:**
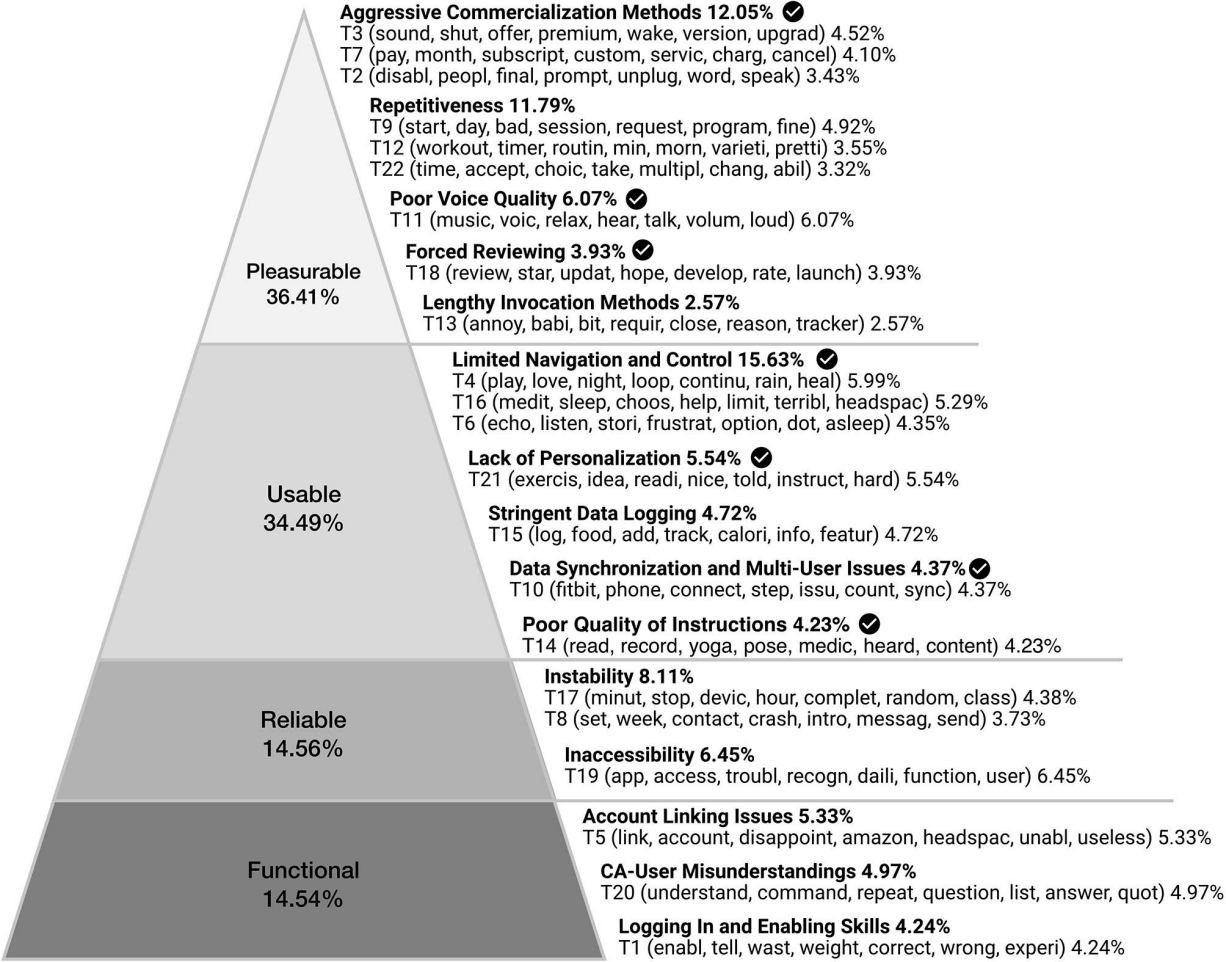
Subjects of criticism of Alexa Skills designed to support wellbeing, grouped within Aarron Walter's hierarchy of user needs (30). Each subject of criticism contains one or multiple topics [T#] generated by STM. The words in parenthesis are the 7 highest probability words of the respective topic. The percentages show the proportion of the reviews. Check mark indicates the topics that have been discussed in prior work by Shin et al. (34).

Following the coding process described in section 3.3.1, we arrived at 15 subjects of user critique which aligned with Aarron Walter's hierarchy of user needs (30), highlight key areas of user concern and critique in relation to the design of the state-of-the-art CHAs experience (see Figure 3 and Supplemental Material). This analysis reveals the highest proportion of users' critiques as pertaining to the pleasurable aspects of Alexa Skills designed to support health and wellbeing, followed by usable, reliable, and functional factors—starting points for design we next examine in greater detail.

### Critiquing Functionality of the CHAs

In order to provide some intrinsic value while also meeting users' needs, any system or interface must first and foremost prove functional (30). Examining reviewers' critiques of CHAs in relation to their functionality can thus provide insight into these essential aspects of their design. Reviewers' critiques in this regard, as surfaced by our STM and comprising 14.54% of the reviews in our corpus, pertained primarily to technical concerns, including issues related to linking third party accounts to the system (5.33%), CAs' capacity to understand users' utterances (4.97%), and enabling and logging in to Skills (4.24%).

#### Account Linking

A number of Skills allow users to link existing online profiles (e.g., Facebook, Google, or Amazon) in order to provide more personalized content. The Fitbit Skill, for example, requires users to connect to their Fitbit account *via* the Alexa Skill in order for users to be able to retrieve their existing data. Several reviews mentioned that users could not link their accounts, and, therefore, were unable to use the system. In some instances, reviewers commented that the Skill had not linked to their account despite system confirmation; “*I linked my account successfully and got a confirmation email, but whenever I ask Alexa anything about Anthem she says ‘account is not linked'. Cannot even begin to use it”* [T5].

These issues not only impacted users' ability to use the technology but also affected their broader perceptions of the system and vendors. A Skill requesting the additional creation of an account despite users' linking of a social media account generated frustration for one reviewer and caused them to question the CA's intentions; “*Very frustrated…How much of my private information do you actually need? I don't think I should need a separate login at all, but certainly after I've given my Amazon and Facebook, you should not be asking for more!”* [T5]. Others simply disabled Skills when unable to gain access without first logging in; “*Link account? What account? Can't Login. Disabled!”* [T5].

#### CA–User Misunderstandings

CAs' capacity to understand users' utterances is a fundamental component of their value contribution, and base functionality. Speech recognition technology, in many regards still in a nascent phase, plays a crucial role in this respect, and many reviews understandably reflected users' dissatisfaction with certain Skills due to their inability to understand their replies.

Reviewers' comments also suggested however that these limitations in functionality were not only tied to fundamental limitations of the technology but to inadequate conversational interaction design also. One reviewer criticized a Skill designed to assess users' health for not understanding simple symptoms despite formulating their response in numerous different ways; “*Very poor implementation. No matter how I stated my symptoms, it kept saying it didn't have an answer”* [T20]. Certain reviewers also commented that they expected to be able to hold meaningful conversations with Skills, and that Skills which could not engage users in such interactions were in turn considered “useless;” “*Asks your name, I give it, it says Hello and asks how I'm feeling and when I respond it repeats itself. Useless Skill”* [T20]. Other reviewers even complained that some Skills could not understand simple utterances—including “Yes” and “No”—and were soon deactivated; “*I've tried endless variations of ‘yes,' ‘let's get started,' ‘go,' ‘okay,' etc. Nothing seems to work. So, this Skill was quickly deactivated”* [T20].

#### Logging in and Enabling Skills

In order to begin using CA Skills within the context of the Amazon ecosystem, users typically have to first find and enable the Skill *via* the Amazon Alexa Skill Library accessed by either mobile or web application. And many reviews reflected problems with this process. In particular, reviewers often mentioned that despite enabling Skills *via* the mobile app, they would be unable to use the Skill and were repeatedly asked to enable it again, e.g., “*Enabled the workout, but keeps telling me to enable the app”* [T1]. One reviewer provided a detailed description of a specific bug encountered while enabling a Skill, in text formatted to reflect their felt frustration; “*I click enable and the text button goes gray. 5 s later the enable comes back from being grayed out. i press enable Skill again, same thing. will NOT ENABLE”* [T1].

Once enabled, certain Skills then require users to create an additional account, and to log in using their credentials—another pain-point for several reviewers who commented that despite using the correct credentials were unable to log in. One reviewer noted that they had even verified their credentials with the vendor yet still could not log in; “*I've tried over a dozen times to get this thing to work and it keeps telling me my information is wrong. It is not wrong I have even verified it with the pharmacy and it still won't work”* [T1].

### Critiquing Reliability of the CHAs

Reliable systems are additionally stable, dependable, and perform consistently, establishing trust, driving user engagement, and making positive experiences possible in the process (30). Representing 14.56% of the reviews contained in our corpus, reviewers' critiques pertaining to the reliability of Skills related primarily to issues of instability (8.11%) and inaccessibility (6.45%).

#### Instability

A number of reviewers commented that Skills would often stop abruptly without any notification. This was particularly frustrating in the case of Skills designed to assist in activities with multiple sequential steps such as fitness and training; “*Midway through the workout it stops and goes silent”* [T17]. Such unexpected behavior confused users and left them with the impression of a general lack of reliability; “*Sometimes it will play the whole night, sometimes it goes silent after just a few minutes. Never know what you're gonna get…”* [T17].

Several reviewers additionally provide comments to indicate that they had identified particular bugs and causes of these events, even reporting these problems to vendors, yet were left without solutions; “*I submitted a report but nothing has happened. The Skill still crashes in the same spot”* [T8]. Another reviewer attempted to solve a similar issue by enabling and disabling the Skill to no effect; “*I've disabled and re-enabled the Skill, but it still crashes at the same point every time I use it. Who can I contact?”* [T8].

#### Inaccessibility

When a Skill proves unavailable, Alexa alerts users with the phase “*Sorry, I'm having trouble accessing your [Skill's name] right now”* [T19]—a source of consternation for many reviewers who commented on the sometimes sporadic availability of certain Skills, and at times questioned whether Skills had even been tested prior to publishing; “*She says she's having trouble accessing this Skill. Was it tested?”* [T19]. Encountering this issue during interaction with a Skill proved particularly frustrating for reviewers who interpreted this as a waste of their time leaving them unable to complete tasks; “*The app doesn't work. If you say ‘how many calories in...' it'll ask for clarification: do you want fat, sugar, energy. Then when you answer, it tells you that it is having trouble accessing the app. Waste of time”* [T19]. One reviewer went as far as to advise potential users not to enable a Skill, claiming that it was not ready for public use; “*Don't waste your time, this app doesn't work…Nothing seems to work, this app is not ready for public use”* [T19].

### Critiquing Usability of the CHAs

The usability of a system relates to both its ease of use, and the ease with which a user can learn to perform basic tasks without following a steep learning curve (30). 34.49% of the reviews in our corpus criticized Skills' usability in terms of limited navigation and control (15.63%), a lack of personalization (5.54%), stringent data logging (4.72%), data synchronization and multi-user issues (4.37%), and the poor quality of instructions (4.23%).

#### Limited Navigation and Control

Many reviews expressed users' discontent with the limitations of certain Skills, most often in terms of the options provided for navigation and control, including the ability, or lack of, to loop, select, skip, or resume content.

These features were particularly pertinent to Skills presenting long audio tracks, a popular application type. Several such systems were critiqued for failing to enable looping; “*This fan sound is great. Only downfall is it only offers 1 hour with no ability to loop or play it all night”* [T4]. And others for preventing users from making selections; “*Love the app. Love sleepcasts. Hate that it randomly plays different ones and doesn't let you easily play just your favorite”* [T4]. These navigational constraints frustrated users greatly, forcing them to listen to content they did not like;

“*It is very frustrating when a story begins and you don't like the voice and say, ‘1Alexa switch stories'..nothing. ‘Alexa change stories'…nothing. You must say, ‘Alexa stop' and start the whole long introduction, categories, etc. thing over again. You can't even reply before the entire list of options has been read. After a few times of these exhausting machinations, I'm ready to quit the whole thing. Too bad because the original story I heard was great.”* [T6]

In other cases, users were forced to listen to the same content repeatedly as they could not ask the Skill to skip content; “*I like the idea of it, and the stories are great, but it keeps playing the same story consecutively. I wish there was a way to skip to the next story. I've been listening to the same story for several nights in a row”* [T6].

#### Limited Personalization

Many reviewers noted the importance of CA customization in relation to the ability to meet their personal health goals. One reviewer contrasted a physical fitness Skill with a mobile app, and suggested allowing users to alter activity timings or create personalized routines to suit their own fitness levels; “*…I know some iOS and android equivalents let you change exercise and rest intervals. They can also provide alternative exercises using the timing system, or even let you make your own routines”* [T21]. Another reviewer suggested that health and fitness Skills might tailor exercises based on a user's age, for example; “*I asked for low impact and this was too hard for me, though I still did what I could do. I'm almost 70 and though I'm ready to exercise and be healthier the workout needs to suit my age”* [T21].

Other reviewers requested new or modified features, such as stretches for particular body parts, and when Skills could not offer such options, stopped using them; “*I also had trouble getting the program to give me a series of stretches for a particular body area, such as upper back. It gave me a shoulder stretch, a chest stretch and a quad stretch, so I stopped it”* [T21].

#### Stringent Data Logging

Another popular genre of Skills allows users to log health and wellbeing data, many eliciting unique forms of critique from users. Many reviewers commented that such Skills were often limited in terms of the types of data they could log, and in the ability to modify logged data. Several reviewers desired for fitness tracking Skills to also enable tracking of food and water intake for example, “*It would be great to log food and water!”* [T15], while others found nutrition tracking Skills overly complicated when it came to logging custom food; “*Has a lot of nice features but it is a REAL PAIN to try and add items to the Custom Foods. Once it has been added it is there and the calories, etc. cannot be changed and the item cannot be deleted. Seems like editing your Custom Foods would be a BASIC feature!!!”* [T15]. Others noted that these Skills often logged calories inaccurately, which they stressed was of paramount importance to the usability of such CHAs; “*Despite trying multiple ways to name the food I wanted it to log, giving it more or less information, it does not log calories correctly. An inaccurate calorie tracker is completely useless”* [T15].

Logging data through voice interaction can quickly deteriorate into a long and drawn-out process, as systems may need to confirm entries while also providing feedback to users. One reviewer proposed alternative methods of confirmation and suggested that CHAs only provide feedback when requested by users in order to keep these interactions brief, and therefore usable;

“*…each time I add something, it tells me how many calories I have remaining for the day. So if I want to add 5 breakfast items in succession, I have to listen to her telling me how many calories I have remaining. I would prefer it if she would only tell me that information when I ask her. After I add something she should just say 'added' or even better just beep. or say '62 added'. As brief as possible.”* [T15]

#### Data Synchronization and Multi-User Issues

Growing adoption of smart speaker devices is additionally reflected in the emerging use of Alexa Echo smart speakers for interaction with external devices or services—a link established with the help of bespoke Skills. In addition to the issues associated with the functionality of account linking discussed in section **??**, several reviewers criticized health monitoring device-related Skills (e.g., Fitbit) in particular for data synchronization and multi-user usability problems.

Reviewers commented that the need to employ a mobile app in order to sync data from an external device to a Skill was counter-intuitive in nature, as such data was often already and more readily, available through the mobile app itself; “*Pulls information from Fitbit app & not your actual Fitbit. You have to open the app, let it sync, don't look at the stats, then ask Alexa to retrieve the information. I'm better off just looking at my watch”* [T10]. Others highlighted the need for such Skills to support multiple devices within a household, as it is often common for a family to own more than one device; “*Only supports one Fitbit profile. We have 3 Fitbit users in our house. This cannot be a rare use case!”* [T10]. One reviewer commented sanguinely “*Alexa isn't advanced enough really to make this worthwhile. If it did connect directly, you can only have one Bluetooth connection, so it there always be trade-offs”* [T10].

#### Poor Instruction Quality

While health and fitness Skills come in many diverse forms, many serve as guides for physical or cognitive activities, the quality of which was a frequent source of critique among reviewers. One reviewer, a novice yoga student, for example commented that instructions were insufficiently detailed to enable them to follow poses;“*The instructor does not describe how to do every pose and sometimes when she does its too fast for me to process before the next move”* [T14]. Another, expert, user of a similar Skill commented, on the other hand, that instructions provided were overly detailed in nature, and suggested presenting different instructions for different groups of users; “*…if you actually want to do yoga there is absolutely no reason for a 20 sec description of how to do mountain pose LOL”* [T14]. Others commented that instructions provided in audio-form were often simply confusing, suggesting a need for visual cues; “*This was too difficult to follow. Maybe because there was no visual, I found the commands confusing”* [T14].

These findings highlight an additional level of complexity in the design of CHAs required not only to hold engaging conversations but to provide precise instructions to users not available in visual form.

### Critiquing Pleasurability of the CHAs

Systems which are pleasurable are additionally able to both delight users and establish lasting relationships (30). Representing 36.41% of the reviews contained within our corpus, we find the pleasurability of users' CHA experiences to be negatively impacted by aggressive commercialization methods (12.05%), repetitiveness (11.79%), poor voice quality (6.07%), forced reviews (3.93%), and lengthy invocation methods (2.57%).

#### Aggressive Commercialization Methods

Reviewers of Alexa health and fitness Skills often commented that commercialization methods negatively impact otherwise pleasurable CHA experiences. Many reviews featured complaints concerning annoyingly frequent subscription prompts, erroneous subscription methods, and inefficient cancellation processes.

Frequent prompting in particular frustrated users, who commented, for example, that “*Everything you do is followed by 15 s of promoting the upgrade to pro membership. you have to explicitly say no every time”* [T2], and that “*Once you indicate you aren't interested, it should disable these prompts. Very frustrating”* [T2]. This led several reviewers to state that they no longer wished to engage with Alexa Skills until such frustrating upselling practices were removed; “*Its so frustrating that I don't even want to deal with this app any more. If there was a way to get her to stop trying to upsell me I might keep it, but right now its a hard pass”* [T3]. In other cases, certain Skills would suddenly limit users from accessing previously free content without prior notification, which users highlighted as frustrating and demotivating experiences; “*It used to be free but with no warning you can't access any of the sounds without paying. Some type of warning would have been great”* [T3].

Additionally, several reviewers reported encountering errors during the process of subscribing to paid Skills, and described unsubscribing as unnecessarily difficult. Others commented that certain Skills related to sleep had charged fees without any notification, and they were unable to figure out how to cancel such unintended subscriptions; “*Asked me if my kids wanted to hear a bedtime story. Now all of a sudden I see I'm going to be getting a monthly charge. I still haven't figured out how to cancel this”* [T7]. This discrepancy in the ease of subscribing and unsubscribing from services was considered by some as deceptive practice; “*If Alexa can automatically start subscriptions for you, she had better have the capability to cancel them just as easy!”* [T7]. One reviewer argued that Amazon should play a more active role in the oversight of the marketplace, mandating, for example, the disclosure of certain practices;“*This kind of tactic should be restricted by Amazon, requiring to disclose any subscriptions or charges PRIOR to enabling any Skill that has such charges”* [T7].

#### Repetitiveness

Many health and fitness Skills target behavior change; a motivation equally often expressed by reviewers for engaging with these systems. And the frequently repetitive nature of CHA content was therefore often in turn critiqued as causing their motivation to fade; “*…don't see myself being motivated to keep using if it never changes”* [T12]. Many reviewers mentioned that Skills designed to support fitness and training in particularly often would not save information relating to prior sessions, meaning that users would be offered the same training sessions each time they opened the Skill, and creating the perception of a lack of variety; “*I just did the 20-minute session for the 4th time. While I like the session with Sarah Beth, I was surprised and disappointed that I got the same exact session each and every time. Add some more variety and it would be great!”* [T9].

#### Poor Voice Quality

Skills designed to guide users through meditation, sleep and yoga practices, were often criticized in particular, for inconsistent volume levels, poor music mixing, rapid pacing of speech, and other voice characteristics including tone and accent.

Many reviewers commented that poor mixing of a narrator's voice and music detracted from these experiences; “*Good but couldn't hear her very well as the music was too loud. Her voice also dropped down in volume at end of each line”* [T11]. When the pace of a narrator's speech was on the other hand too fast for a Skill designed to support meditation, reviewers complained of experiencing the opposite of the desired effect; “*…she talked WAYYYYYY to (Sic) fast to be relaxing or to even be able to do what she was saying. Not relaxing at all”* [T11]. Others commented that Skills often employed voices which did not match a Skill's content or otherwise failed to appeal to users; “*Seriously, the voice gave me the creeps. Going to sleep with a melodic, soothing, relaxing female voice was what I thought it would be. It was horrible! A weird, scratchy creepy voice…yuck!”* [T11].

#### Forced Reviewing

Several Skills contained within this sample required users to provide five-star reviews in order to be able to use the Skill for free—an unscrupulous practice often, although not always, derided by reviewers; “*Would be good if it didn't beg for five star reviews. I'm concerned many of these ratings are artificial”* [T18]. While many Skills were otherwise made available to users for free, frequent prompting to provide reviews was often perceived as harassment by reviewers, who on occasion provided negative reviews as a result, and chose not to use these Skills; “*I LOVED this Skill…until they started hassling me to review, buy more sounds, review Buy More Sounds REVIEW…here's your review. Now I'm going to disable this Skill go find something else that wont harass me out of the relaxed state it put me in”* [T18].

#### Lengthy Invocation Methods

To launch an Alexa Skill, users must invoke that particular Skill by voicing a predefined phrase; either by (1) combining the Skill's name with a question or command (intent) (e.g., “Alexa, ask [name of the Skill] for my heart rate.”), (2) mentioning the Skill's name without a specific question, request, or command (no intent) (e.g., “Alexa, open [name of the Skill].”) following which the user must then wait until the Skill has been invoked to ask their question, or (3) by asking Alexa to perform a task without naming the Skill that should fulfill that request, e.g., “Alexa, what's my heart rate?”^8^

Several reviewers complained about the need for lengthy invocation phrases or to invoke individual Skills before every interaction. One reviewer, who happened to be a new parent, commented that a Skill's transition from a name-free to intent-based invocation method had resulted in an inefficient and frustrating experience, adversely impacting their already stressful lived experience; “*…this was so much better before the recent change. Whoever made that change clearly doesn't understand how stressed, sleep deprived and short on time new parents are. Every little efficiency helps when you have a baby so I really hope they rethink this change and go back to how it was before they F'ed it up :(”* [T13].

When Skills provided no other method to invoke the Skill other than the “no intent” invocation method while also requiring users to invoke the Skill prior to each interaction, reviewers often reported giving up on a Skill as a result of this lengthy turn-taking process; “*When using this Skill you have to tell Alexa to open medicine tracker for every entry, even if they are back to back. Very annoying. I gave up”* [T13].

Having identified topics of reviewer critique key to the experience of functional, reliable, usable and pleasurable CHA experiences, we next turn to reflect on the implications of these findings for design.

## Discussion

Adopting the STM approach presented in this article enabled us to produce an effective overview of the Alexa health and fitness skills design space. This mapping both pinpoints popular genres of CA at the present point in time, and highlights opportunities to support health and wellbeing through previously under- and un-explored CA applications. Our results show in particular an overwhelming prevalence of Skills designed to support meditation and fitness training. These Skills often employ music as their primary form of content—in fact the most common use case for smart speaker devices [i.e., streaming music (54, 55)]. This finding also reflects however a conservative assessment of the CA medium's potential to support health and wellbeing; which extends beyond purely transactional inquiries and the instruction of meditation and fitness activities.

Designing CAs for more complex and conversational use cases (e.g., health monitoring and tracking, providing social support etc.) is of course not without its challenges, as highlighted in the findings of this review of reviews, as in prior work (56). Overcoming these outstanding technical, ethical and design challenges requires developing insight into users' experiences of these still-emergent systems, as we undertook to examine in this study by means of analysis of 2741 critical reviews of 485 Alexa health and fitness Skills; identifying 15 subjects of reviewer critique key to the experience of functional, reliable, usable and pleasurable CHA experiences. We further reflect on these findings in light of our research questions.

### Users' Critiques on the CHAs

Our first research question pertained to exactly those subjects of critique, as means of insight into reviewers,' and in turn users,' experiences. Aligning these topics with Arron Walters' hierarchy of user needs, we found a higher proportion of reviews pertained to design concerns of pleasurable and usable experiences than to reliability and functionality.

One interpretation of these results is as reflecting users' strong desire to interact with CHAs despite limitations in regard to their functionality and reliability—striving to engage actively in usable and pleasurable experiences—and, in turn, reflecting the value users associate with CHAs as means to support wellbeing. This interpretation aligns with the findings of prior work. For example, Kocielnik et al. (57) reported that many users in their own study looked past the current technical limitations of CAs, expressing the potential value of a dedicated voice-based conversational modality for self-reflection found personal, interactive and engaging despite technological constraints. It may also be hypothesized that many of the Skills referred to by the reviews included within this study employ transactional interactions of a kind already supported by these CA systems despite their limitations.

Prior literature additionally underlines, despite these limitations, that there is much appropriate interaction design efforts can do to create improved experiences for CHA users (21–25). The primary focus of users' reviews on concerns of usability and pleasurability not only corroborates these prior findings but also provides us with several starting points for these efforts.

### Implications for Designing CHAs

Drawing on the findings of this study, we are able to identify a set of implications for the design of functional, reliable, usable and pleasurable CHA experiences extending prior work. These implications for design pertain not only to individual Alexa Skills, but also to the Alexa Device Ecosystem as a whole, and broader Amazon Marketplace.

#### For Functional CHA Experiences | Keep Setup Processes Simple, Facilitate Connectivity, and Prepare Meaningful Responses to Predictable Queries

Our findings emphasize the importance of keeping the Skill setup process (Skill enabling and Log in) as simple as possible while also facilitating connectivity across the Skill ecosystem and peripheral devices, in order to prevent user frustration. Many CHAs allow users to link their existing social media accounts as means of facilitating this process and enabling personalized content and experiences key to the health and wellbeing context. Our results additionally suggest however that careful implementation of these features is essential to gaining users' trust, as complex setup processes requiring the provision of excess information may not only frustrate reviewers but also lead users to question the CHA's intentions. Furthermore, our results suggest the need to engage users in meaningful interactions by responding to predictable queries with logical and effective responses. Meaningless and repetitive fallback responses to queries, in contrast, often led users to consider Skills useless, and to abandon their use.

Failure to fulfill these foundational needs can have a significantly adverse impact on the overall CHA user experiences. We, therefore, suggest keeping setup processes simple, facilitating connectivity, and designing for meaningful responses to predictable queries in order to support functional CHA interactions, as a foundation for reliable experiences.

#### For Reliable CHA Experiences | Provide Ancillary Support, and Foster User-Vendor Relationships

Issues raised by reviewers pertaining to CHAs' reliability most often concerned inaccessibility and abrupt crashing of the system. A Skill can prove unavailable for many reasons, from hardware failures to software bugs, and even errors within users' own device and profile settings (e.g., incorrect profile and inadequate user permissions).^9^ While the designers of CHAs may not have the capacity to address all of these issues and limitations, they can transparently inform users about potential problems and provide relevant information to support troubleshooting.

Other problems leading to abrupt crashing of CHAs, however, reflect the need for rigorous testing of Skills prior to and following their release. As reviewers' comments often reflect, it is also essential that CHA vendors provide ancillary support in case users do encounter such issues. We found that some reviewers had tried to reach out to vendors to report such issues, yet were not able to receive support, and therefore unable to use the Skill. Prior work has suggested that CHAs could function as a direct route to connecting with vendors in order to obtain up-to-date and reliable information (34), and facilitating such relationships requires continuous support and communication with users—which this current study found to be lacking within the present CHA landscape.

In addition to rigorous testing of CHAs, we, therefore, suggest making ancillary support available to users while also fostering positive relationships between users and vendors of these emerging technologies as means to creating reliable CHA interactions, as make usable experiences possible.

#### For Usable CHA Experiences | Support Navigation and Personalization Features

Comments from many of the reviews across this sample expressed a lowered sense of control and freedom on users' behalf, often in relation to the limited navigation features (e.g. back, loop, pause, and skip) made available to them. The absence of such relatively trivial navigational controls, led reviewers to consider many CHAs useless, particularly those providing step-by-step guidance, content in episode form, or playing music. These findings align with prior research which suggests that a lack of control and freedom tends to lead users to reduce their interaction over time and gradually abandon CHAs for activities other than those they believe agents capable of performing easily (58). Reviewers in this study also experienced a lack of control in relation to the types of data they were able to log, the lack of ability to edit data, and in unsubscribing from accidental or erroneous subscriptions.

In addition to these features deemed essential to CHAs' usability by users, reviewers also expressed a desire for means of tailoring systems to match their personal health goals. We observed that many Skills provided standardized content which did not work for users across different age groups, physical abilities and levels of expertise. As a result, users were unable to use certain Skills despite their strong desire to do so. These findings highlight the need to design inclusively, in order to maximize usability for all users.

We therefore suggest supporting basic and flexible navigation and control features in addition to enabling personalization in support of usable CHA experiences capable of supporting pleasurable interactions.

#### For Pleasurable CHA Experiences | Prioritize Brevity, Provide Variety, Approach Voice as Design Material, and Commercialize Ethically

Many of the reviews across this sample highlighted the importance of brevity in interaction, as paramount to pleasurable CHA design. Our findings suggest that it is important to consider concise and intuitive invocation phrases as the critical entry points, in particular, for engaging and pleasurable user experiences. Reviewers represented in this study often preferred the ‘name-free' method of invocation which saved time and effort by negating the need to invoke the system by repeatedly calling its name every time they wished to interact. Such requirements were highlighted by users as particularly important in the design of CHAs given users' health conditions or otherwise complex contexts.

In addition to expressing annoyance in relation to the need to repeatedly invoke CHAs, reviewers also spoke of the repetitive nature of CHA responses to queries as frustrating. In the cases of fitness and training agents, repetitive content additionally demotivated reviewers from pursuing positive health-related behavior change—suggesting the need to provide a wider variety of content in order to motivate and support long-term CA–user engagement.

In line with many prior findings, our results additionally emphasize the role of voice characteristics, including consistency of volume levels, the pace of speech, tone, and accent, in engaging users in pleasurable CHA experiences (20, 34). We observed that these voice features were considered especially important in the case of those Skills designed to support meditation, sleep, and yoga—the most prevalent type of CHAs.

Finally, our results underlined how the intrusive nature of various commercialization practices negatively impacted the pleasurable nature of many users' CHA experiences; through aggressive prompting to subscribe, purposive obstacles to unsubscribing, or forcing positive reviews in return for free use (59). These findings highlight the importance of finding creative ways of commercialization which value users' engagement and the onus of developing ethical monetization practices.

We therefore recommend prioritizing brevity and providing variety in interaction design, weighing voice as design material, and pursuing ethical means of commercialization in order to create functional, reliable, usable and pleasurable CHA experiences.

### Learning From Reviews

Finally, we reflect on the value of this method itself as a means of generating insight, for design, from reviews, of users' experiences. We employ in this instance an automated approach, which in comparison to the manual review of reviews, as previously employed in relation to Alexa Skills in particular (34), has certain advantages in terms of efficiency and scale, while also yielding insight of value for design. This article provides several examples of the ways in which individual reviews can highlight opportunities for new features, identify bugs, highlight users' pain points and provide rich insight into users' emotional experiences and broader social context. This work also shows how the aggregate analysis of reviews can additionally facilitate an informative mapping of the CHA design space, likewise highlighting under- and un-explored design opportunities and technology framings.

Online reviews are of course on the one hand subjected to a variety of possible biases, and may be considered somewhat removed from users' experiences; a less rich and emotionally-informative source of insight. They are however also often very much situated in users' real-world experience, made voluntarily, and expressed in users' own word- –allowing and promoting unconstrained expression. The automated analysis of reviews is therefore, it may be argued, in many ways closer to Ecological Momentary Assessments of representative user experiences than many other design research methods. This is evidenced for example in the insight additionally gained from the emotional weight often communicated *via* reviewers' use of capital letters and excessive use of punctuation marks.

By examining critical review in particular, we have been able to highlight users' pain-points, as well as critical opportunities for improving the state-of-the-art CHA experience. Continued adoption and future exploration of this still-nascent approach to understanding technology may itself over time further our capacity to generate insight into users' experiences of technology use, in support of the design of experiences functional, reliable, usable, and pleasurable in nature.

## Limitations

The data included in this review of reviews is limited to those Amazon Alexa skills published in the “health and fitness” category. There may, however, be other health-related skills published in other categories (e.g., “smart home,” “food and drink”) which we have therefore not included. We additionally relied primarily on vendors' descriptions in order to classify skills into different categories. It is possible however that these descriptions may not accurately represent skills' actual features as they are modified and updated.

As seen in Figure 2, a large number of the reviews analyzed during this study pertained to meditation, fitness and training activities. Our results and design implications may therefore be biased toward such CHAs. Online reviews are additionally subject to self-selection biases. For example, reviewers encountering an extreme experience, either positive or negative in nature, are more likely to review a product than those with more moderate views and experiences (60, 61). In addition, the results of this study are limited to those critiques which reviewers chose to mention; and there may therefore exist other critiques of these systems which this study was unable to capture.

Lastly, while the probabilistic model of STM is an innovative approach to investigating a large number of online reviews, by generating the topics based on word counts, some of the context and meaning of these topics might have been lost.

## Conclusion

Based on the automated topic modeling of 2741 critical reviews of 485 Alexa Skills across 19 health and fitness categories, this work contributes (i) an understanding of critical factors affecting users' experiences of CHAs, (ii) recommendations for the future design of effective and engaging CHAs, and (iii) a novel approach to the critical analysis of online reviews in support of design implications. Reflecting on the 15 subjects of criticism identified across these key areas of design, we present implications for the design of CHA experiences rendered; (i) functional by keeping setup processes simple, facilitating connectivity, and preparing meaningful responses to predictable queries; (ii) reliable by providing ancillary support, and fostering user-vendor relationships; (iii) usable by supporting navigation and personalization features; and (iv) pleasurable by prioritizing brevity, providing variety, approaching voice as design material, and commercializing ethically.

## Data Availability Statement

The original contributions presented in the study are summarized in the article/Supplementary Material, further inquiries can be directed to the corresponding author/s.

## Author Contributions

RM and DR analyzed the data with the assistance of KD. RM and KD contributed to writing the manuscript. All authors contributed to the final review and editing and have approved the final manuscript.

## Funding

This project was supported by the Novo Nordisk Foundation, Grant Number NNF16OC0022038, and the Copenhagen Center For Health Technology (CACHET).

## Conflict of Interest

The authors declare that the research was conducted in the absence of any commercial or financial relationships that could be construed as a potential conflict of interest.

## Publisher's Note

All claims expressed in this article are solely those of the authors and do not necessarily represent those of their affiliated organizations, or those of the publisher, the editors and the reviewers. Any product that may be evaluated in this article, or claim that may be made by its manufacturer, is not guaranteed or endorsed by the publisher.

## Footnotes

^1^ https://www.amazon.com/alexa-Skills/b?ie=UTF8&node=13727921011

^2^ https://www.amazon.com/dp/B07JJNX41X

^3^ https://www.amazon.com/dp/B07F659541

^4^ https://www.amazon.com/dp/B084KW2N2V

^5^ https://rdrr.io/github/bstewart/stm/man/topicQuality.html

^6^STM's labelTopics function generates a set of words (Top Words) describing each topic.

^7^STM's findThoughts function outputs a given number of highly associated documents for a particular topic.

^8^ https://developer.amazon.com/en-US/docs/alexa/custom-skills/understanding-how-users-invoke-custom-skills.html#cert-invoke-specific-request

^9^ https://www.amazon.com/gp/help/customer/display.html?nodeId=G62ZU6D99SYG3BWU
