# Supplementary material for: What's Up With These Conversational Health Agents? From Users' Critiques to Implications for Design
Source: Front Digit Health. 2022 Apr 7;4:840232. doi: 10.3389/fdgth.2022.840232 (PMC9021431; doi:10.3389/fdgth.2022.840232)
Supplement: Supplementary file 1 [file Table_1.pdf]

Supplementary Material

Taxonomy of critical reviews of Alexa's health and fitness Skills. Each topic (T#) is categorized into one of the 15 subjects of criticism. These subjects of criticism are further grouped into four key areas of users' needs. P = topic proportion.

T#	Topic Label (Highest Probability Words)	P
	**PLEASURABLE**	**36.41%**
	**Aggressive commercialization methods**	12.05%
3	Up-selling practices (sound, shut, offer, premium, wake, version, upgrad)	4.52%
	"I've disabled this skill because it is so irritating that it defeats the purpose of listening to relaxing sounds. I shouldn't have to say more than once that i don't want to hear more about the premium membership."	
7	Difficult to cancel subscription (pay, month, subscript, custom, servic, charg, cancel)	4.10%
	'If Alexa can automatically start subscriptions for you, she had better have the capability to cancel them just as easy!"	
2	Frequent subscription prompts (disabl, peopl, final, prompt, unplug, word, speak)	3.43%
	'Everything you do is followed by 15 seconds of promoting the upgrade to pro membership."	
	**Repetitiveness**	11.79%
9	Repetitive sessions (start, day, bad, session, request, program, fine)	4.92%
	"If I pause it I have to start all over with introduction."	
12	Lack of content variety (workout, timer, routin, min, morn, varieti, pretti)	3.55%
	'Pretty good, but it's the exact same workout every time. I thought there would be more variety."	
22	Repetitive interaction (time, accept, choic, take, multipl, chang, abil)	3.32%
	"Every time my wife gives the command, Alexa says an adult needs to give permission. I have given permission countless times, but it always reverts back."	
11	**Poor voice quality** (music, voic, relax, hear, talk, volum, loud)	6.07%
	'Voice is too soft and at the same time music is too loud."	
18	**Forced reviewing** (review, star, updat, hope, develop, rate, launch)	3.93%
	"While I do enjoy the app, I don't appreciate it trying to force a 5 star rating."	
13	**Lengthy invocation methods** (annoy, babi, bit, requir, close, reason, tracker)	2.57%
	"Having to say the entire extra phrase every single time of 'Alexa, ask Baby Connect to...' is very inefficient and frustrating especially when we got used to it without the extra phrase.	
	**USABLE**	**34.49%**
	**Limited navigation and control**	15.63%
4	No loop function (play, love, night, loop, continu, rain, heal)	5.99%
	"This fan sound is great. Only downfall is it only offers 1 hour with no ability to loop or play it all night."	
16	Can't choose specific content (medit, sleep, choos, help, limit, terribl, headspac)	5.29%
	'I've had over an hour of meditation and now the skill keeps playing one or three minute meditations. I'd prefer to be able to choose 7-10 minute ones."	
6	Poor navigational structure (echo, listen, stori, frustrat, option, dot, asleep)	4.35%
	"It is very frustrating when a story begins and you don't like the voice and say, 'Alexa switch stories'..nothing. 'Alexa change stories'...nothing. You must say, 'Alexa stop' and start the whole long introduction, categories, etc. thing over again."	
21	**Lack of personalization (5.54%)** (exercis, idea, readi, nice, told, instruct, hard)	5.54%
	'... it would be nice to be able to configure some of the parameters. I know some iOS and android equivalents let you change exercise and rest intervals."	
15	**Stringent data logging** (log, food, add, track, calori, info, featur)	4.72%
	"It does not log calories correctly. An inaccurate calorie tracker is completely useless "	
10	**Data synchronization and multi user issues** (fitbit, phone, connect, step, issu, count, sync)	4.37%
	"Pulls information from Fitbit app and not your actual Fitbit. You have to open the app, let it sync, don't look at the stats, then ask Alexa to retrieve the information. I'm better off just looking at my watch."	
14	**Poor quality of instructions** (read, record, yoga, pose, medic, heard, content)	4.23%
	'This is definitely not for beginners. I'm fairly new to yoga, but this was really difficult to follow."	
	**RELIABLE**	**14.56%**
	**Instability**	8.11%
17	Abrupt Stop (minut, stop, devic, hour, complet, random, class)	4.38%
	"It keeps skipping and stopping mid-exercise saying 'I'm not sure what went wrong'.'	
8	Crash (set, week, contact, crash, intro, messag, send)	3.73%
	"Used to work every time I started, but for the past few months, the skill crashes every time... "	
19	**Inaccessiblility** (app, access, troubl, recogn, daili, function, user)	6.45%
	"She says she's having trouble accessing this skill. Was it tested?"	
	**FUNCTIONAL**	**14.54%**
5	**Account linking issues** (link, account, disappoint, amazon, headspac, unabl, useless)	5.33%
	"Unable to link to my account."	
20	**Conversational Agent (CA)-user misunderstandings** (understand, command, repeat, question, list, answer, quot)	4.97%
	"This app requires responses to questions, but it doesn't seem able to understand responses."	
1	**Logging in and enabling Skills** (enabl, tell, wast, weight, correct, wrong, experi)	4.24%
	"I've tried over a dozen times to get this thing to work and it keeps telling me my information is wrong. It is not wrong I have even verified it with the pharmacy and it still won't work."	
